# Identification and experimental validation of demethylation-related genes in diabetic nephropathy

**DOI:** 10.3389/fgene.2025.1675592

**Published:** 2025-11-26

**Authors:** Hui Miao, Yunke Zhu, Jiaqi Zheng, Chunfeng Deng, Yi Zeng, Fei Tang, Xi Liu

**Affiliations:** Department of Nephrology, Longgang Central Hospital of Shenzhen, Shenzhen, China

**Keywords:** diabetic nephropathy, demethylation, machine Learning, Biomarkers, drug forecasting

## Abstract

**Background:**

Diabetic nephropathy (DN) is a major microvascular complication of diabetes, and its pathogenesis is closely associated with abnormal epigenetic regulation, particularly the silencing of tumor suppressor genes due to hypermethylation of promoter regions. This study was to investigate the workings of demethylation in diabetic nephropathy by applying bioinformatics methods.

**Methods:**

DN-related datasets (GSE142153 and GSE154881) and demethylation-related genes (D-RGs) were included. Differentially expressed genes (DEGs) (DN vs. normal) were obtained. Candidate genes were obtained from the intersection of DEGs and D-RGs. To identify key genes, the Least absolute shrinkage and selection operator (LASSO) and Boruta algorithm, and expression validation were used for screening. The expression validation was used to identify biomarkers. The receiver operating characteristic (ROC) curve was subsequently utilized to assess the biomarkers’ capability to distinguish diseased from normal samples. Subsequently, a predictive nomogram was created to estimate the likelihood of developing DN. In addition, functional enrichment, immune infiltration, subcellular localization, correlation of biomarker expression with renal function, correlation for other diseases, network analysis of molecular interactions and computational drug prediction were carried out. Lastly, Real-Time Quantitative Reverse Transcription Polymerase Chain Reaction (RT-qPCR) was carried out to confirm the expression levels of biomarkers in blood samples.

**Results:**

*CXCL2* and *MLF1* were determined to be biomarkers that exhibited notably elevated expression levels in the DN, in contrast to the normal group. Then the nomogram network was built, which had high prediction accuracy. Pathways most significantly enriched by *CXCL2* and *MLF1* included cytokine-cytokine receptor interaction and MAPK signaling pathway. Five types of immune cells were identified by immune infiltration analysis. In the RNA binding protein (RBP) -mRNA regulatory network, seven pathways were co-enriched in both biomarkers. In the TF-mRNA regulatory network, TFs shared by both biomarkers include JUN, GATA2, and SRF. Finally drug prediction analysis found a total of 172 target drugs for *CXCL2* and *MLF1*. RT-qPCR experiment revealed that both biomarkers showed a notable rise in the DN group relative to the normal group. RT-qPCR results revealed the DN exhibited notably increased expression levels of the two biomarkers (*CXCL2* and *MLF1*) compared to the normal group.

**Conclusion:**

*CXCL2* and *MLF1* were identified as diagnostic biomarkers for DN, offering a new reference for its treatment.

## 1 Introduction

Diabetic nephropathy (DN), a progressive microvascular complication of diabetes mellitus, is characterized by glomerular sclerosis, proteinuria, and renal fibrosis, and it is one of the primary causes of chronic kidney disease (CKD) and end-stage renal disease (ESRD) globally (Sagoo and Gnudi, 2020; Anders et al., 2018). The progression from DN to ESRD is driven by persistent hyperglycemia, chronic inflammation, and renal fibrosis (Yuan et al., 2017; Qiu et al., 2023). In a chronic hyperglycemic environment, epigenetic regulation interacts with renal hemodynamic alterations (glomerular hypertension, hyperfiltration) and metabolic disturbances (oxidative stress, inflammation) to drive renal cell injury and the activation of mesangial cells and myofibroblasts (Amorin et al., 2019; Li et al., 2024). This process ultimately leads to proteinuria and renal failure through glomerulosclerosis and tubulointerstitial fibrosis (Zhao et al., 2024) (Supplementary Figure S1). Epidemiological studies indicate that DN accounts for approximately 40%–50% of ESRD cases globally, with regional variations observed across different populations (Taylor et al., 2019; Yoshida et al., 2017). The increasing prevalence of DN, driven by the rising incidence of diabetes and population aging, significantly contributes to morbidity, mortality, and healthcare expenditures, with annual costs exceeding $100 billion globally (Thomas, 2019; GBD, 2015 Eastern Mediterranean Region Diabetes and Chronic Kidney Disease Collaborators, 2018). The primary risk factors for DN include chronic hyperglycemia, hypertension, and obesity, which collectively induce oxidative stress, inflammation, and renal injury (Jin et al., 2023; Nadaf and Killedar, 2020; O'Connor et al., 2021). While lifestyle modifications and dietary interventions, such as weight loss and glycemic control, may delay disease onset, the progression of DN remains a critical challenge (Sagoo and Gnudi, 2020). The development of DN is driven by a complex array of molecular mechanisms, including oxidative stress, chronic inflammation, dysregulated autophagy, and programmed cell death, which collectively contribute to renal injury and fibrosis (Jin et al., 2023; Nadaf and Killedar, 2020; O'Connor et al., 2021). Emerging evidence suggests that polyphenols, due to their antioxidant and anti-inflammatory properties, may have therapeutic potential in DN. Additionally, sodium-glucose cotransporter 2 (SGLT2) inhibitors have demonstrated renoprotective effects in clinical trials (Jin et al., 2023; Zhou et al., 2022; Knaus et al., 2021). However, despite these advancements, the absence of reliable biomarkers for early detection and risk stratification remains a major barrier in DN management, limiting the timely initiation of targeted therapies and personalized treatment strategies (Ruiz-Ortega et al., 2020). Therefore, identifying novel biomarkers is essential to improve early diagnosis, elucidate disease mechanisms, and facilitate the development of targeted therapies.

One important epigenetic alteration that is essential for controlling gene expression and maintaining genomic integrity is DNA methylation (Deng et al., 2022). In DN, aberrant DNA methylation patterns have been increasingly implicated in disease progression, with hypermethylation of specific genes contributing to renal fibrosis and dysfunction (Quinn et al., 2021). Active DNA demethylation, primarily mediated by ten-eleven translocation (TET) enzymes, plays a crucial role in cellular homeostasis; however, dysregulation of this process has been associated with increased oxidative stress and renal injury (Wang et al., 2022). Recent advances suggest that targeting DNA methylation and demethylation pathways may represent a novel therapeutic approach for DN. DNA methyltransferase (DNMT) inhibitors, such as 5-Aza-2′-deoxycytidine, have been shown to reverse hypermethylation and restore gene expression; however, their clinical application is hindered by toxicity and limited specificity (Fossum et al., 2021). Natural compounds, such as epigallocatechin gallate (EGCG), have demonstrated the ability to modulate methylation patterns, thereby reducing renal fibrosis and inflammation via the NF-κB signaling pathway (He et al., 2022). Furthermore, blood-based DNA methylation signatures are emerging as potential biomarkers for predicting DN progression and may shed light on the early disease processes and identify possible therapeutic approaches (Nguyen et al., 2022). Additionally, the interaction between DNA methylation and key transcription factors, such as AP-1 and TET1, has been shown to regulate critical protective pathways, including the Nrf2/ARE pathway, which plays a vital role in mitigating oxidative stress and inflammation in DN (24). These findings highlight the importance that epigenetic control in the progression of DN and suggest its potential as a valuable target for future therapeutic strategies.

Bioinformatics plays a significant role in type 2 diabetes (T2D) research, providing robust support for the diagnosis and treatment strategies of this disease (Kato et al., 2024; Chen R. et al., 2024).This study identified *CXCL2* and *MLF1* as novel diagnostic biomarkers for diabetic nephropathy through integrated bioinformatics and machine learning approaches, with experimental validation confirming their significant upregulation in patient samples. Functional analyses revealed their involvement in key pathological pathways including immune signaling and cellular stress response, associated with specific immune cell infiltration patterns. Regulatory network analysis uncovered core transcription factors (JUN, GATA2, SRF) co-regulating these biomarkers. Furthermore, 172 potential targeted drugs were predicted, providing new insights for precision diagnosis and targeted therapy of diabetic nephropathy.

## 2 Materials and methods

This section detailed the data sources, analytical methods, and experimental workflow employed in this study, which comprised the following key components: data acquisition and preprocessing; candidate gene identification and analysis; biomarker characterisation; functional enrichment analysis; immune infiltration analysis; regulatory network construction; drug prediction; and experimental validation. The research workflow is presented in Supplementary Figure S2.

### 2.1 Data source

The Gene Expression Omnibus (GEO) (https://www.ncbi.nlm.nih.gov/geo/) was utilized to obtain diabetic nephropathy (DN) related transcriptomic data. Specifically, GSE142153 (GPL6480) included 23 peripheral blood specimens collected from DN (DN group) and 10 from healthy donors (control group) (Li et al., 2021). In addition, GSE154881 (GPL24676) included five samples of blood samples from DN patients (DN group) and five samples of blood samples from healthy individuals (control group) (Zhang et al., 2024). In addition, a search for the keyword “demethylation” via the GeneCards platform (https://www.genecards.org) yielded a total of 3741 demethylation-related genes (D-RGs) (Supplementary Table S1) (Kang et al., 2024).

### 2.2 Identifying and analysing candidate genes

Differentially expressed genes (DEGs) were collected in the GSE142153 (DN vs. normal) and used the package of “limma” (v 3.54.0) (Ritchie et al., 2015) (p value <0.05 and |log2fold change (FC)| > 1). Volcano map and heat map were developed employing the “ggplot2” (v 3.4.1) (Ito and Murphy, 2013) and the “ComplexHeatmap” (v 1.0.12) (Gu et al., 2016), respectively. The intersection between DEGs and D-RGs was used to identify candidate genes, and the “VennDiagram” (v 1.7.1) (Chen and Boutros, 2011) was utilized for draw a Venn diagram to show the result. Finally, Gene Ontology (GO) and Kyoto Encyclopedia of Genes and Genomes (KEGG) were conducted utilizing the “clusterProfiler” (v 4.2.2) (Wu et al., 2021) (a djust P < 0.05). A protein-protein interaction (PPI) network was drawn using the STRING (https://string-db.org/). The threshold was an interaction score ≥0.4.

### 2.3 Identification of biomarkers

Machine learning was employed to identify key genes. First, we got the candidate characterization genes one in the GSE142153 all sample using regression analysis using least absolute shrinkage and selection operator (LASSO) with the “glmnet” (v 1.0.13) (Li et al., 2022) was conducted. Configuration details: nfold set to ten, with the best lambda value is determined using a tenfold cross-validation process. Next, the Boruta algorithm was constructed using the R package “Boruta” (v 8.0.0) (Mahmoudian et al., 2021) to get candidate characterization genes 2. The VennDiagram package (v 1.7.1) was utilized to determine the intersection of the two algorithms (candidate characterization genes one and candidate characterization genes 2), which was denoted as the key genes. The key genes with statistically notable differences were observed. and consistent matched expression patterns in GSE142153 and GSE154881 were used as biomarkers (p < 0.05). The Receiver Operating Characteristic (ROC) curve was used in the GSE142153 using the “pROC” (v 1.18.0) (Robin et al., 2011), and the area under the curve (AUC) value was calculated to evaluate its capacity for accurate diagnosis for DN, with an AUC >0.7. In the GSE142153, the nomogram network was constructed using the R package “rms” (v 6.8.1) (Li M. et al., 2023). In GSE142153, the R package “calibrate” (v 1.7.7) (Sui et al., 2022) was utilized to assess predictive performance through the use of calibration curves. Additionally, in the GSE142153, By plotting the ROC curve based on the nomogram network using the “pROC” (v 1.18.0).

### 2.4 Gene set enrichment analysis (GSEA)

First, the “psych” (v 2.1.6) (Harrison and Goozee, 2014) was employed to determine The Spearman correlation coefficient between each of the biomarkers and the other genes in the GSE142153. The biomarker-gene interactions were then quantified and ranked by correlation coefficients (from big to small). Subsequently, c2. cp.kegg_legacy.v2024.1.Hs.symbols.gmtt have been downloaded from Molecular Signatures Database (MSigDB) (https://www.gsea-msigdb.org/gsea/msigdb/) as background sets. Finally, the R package “clusterProfiler” (v 4.2.2) (Wu et al., 2021) (|NES|>1, a djust p < 0.05) was used for biomarker GSEA analysis. The visualization of the results was achieved by utilizing “enrichplot” (v 1.18.3) (Shi et al., 2022).

### 2.5 Analysis of the immune infiltration

First, the immune richness of 22 immune cells in the GSE142153 between two groups (DN vs. normal) was calculated using the CIBERSORT algorithm (v 0.1.0) (Chen et al., 2018). The Wilcoxon test was employed to evaluate immune cell infiltration differences between the two groups, and the differential immune cells were screened out as differential immune cells (p < 0.05). Box plots were used using the “ggplot2” (v 3.4.1) (Kato et al., 2024). The R package “psych” (v 2.1.6) (Musselman and Kay, 1986) was then employed for conducting Spearman correlation analysis between the differential immune cells and differential immune cells, |(cor)| > 0.3 and p < 0.05. Additionally, the R package “psych” (v 2.1.6) was then employed for conducting Spearman correlation analysis among the differential immune cells and biomarkers, |(cor)| > 0.3 and p < 0.05.

### 2.6 Biomarker function analysis

To gain insight into the organelles where the biomarkers exert their functions, the Hum-mPLoc 3.0 (http://www.csbio.sjtu.edu.cn/bioinf/Hum-mPLoc3/) was used to analyze the subcellular localization of the biomarkers, and the bar charts were drawn using the “ggplot2” (v 3.4.1) for visual presentation. To visualize the chromosomal locations of the biomarkers, the “OmicCircos” package (v 1.38.0) (Zhang et al., 2013) was employed to graphically represent the chromosomal distribution of the biomarkers. To investigate the clinical significance of biomarkers in DN, the correlation between biomarkers and renal function was further analyzed. The biomarker expression level and GFR values in Ju CKD TubInt dataset were downloaded from Nephroseq V5 database (http://v5.nephroseq.org), and Correlation between biomarker expression and GFR values was analysed using Spearman correlation analysis using the R package “psych” (v 2.1.6), with |(cor)| > 0.3 and p < 0.05. The CTD (http://ctdbase.org/) was used to investigate relationships between biomarkers and multiple diseases.

### 2.7 Construction of the network

To analyze the molecular regulatory mechanisms of biomarkers (mRNAs), the Starbase v2.0 (http://starbase.sysu.edu.cn/) was utilized for predicting the targeting linkages between mRNAs and RBPs, and the software Cytoscape (v 3.8.2) (Szklarczyk et al., 2017) was used for the construction of mRNA-RBP regulatory networks. Transcriptional level regulation is an important part of gene expression regulation, in which transcription factors (TFs) realize transcriptional regulation of a gene by binding to specific nucleotide sequences upstream of the gene. The TRRUST database (https://jaspar.genereg.net) of the NetworkAnalyst platform (https://www.grnpedia.org/trrust/) was utilized for TFs prediction of biomarkers, and a network diagram of TF-mRNA interactions was constructed using Cytoscape (v 3.8.2).

### 2.8 Drugs forecasting

To identify potential compounds for the biomarkers using the DGIdb (https://dgidb.genome.wustl.edu/), the targeted drugs that were able to upregulate these two biomarkers and had a supported reference greater than one. And Cytoscape (v 3.8.2) was applied to create a network graph of the biomarkers and all the drugs they predicted.

### 2.9 RT-qPCR

Blood samples were obtained from five individuals diagnosed with DN at Longgang Central Hospital of Shenzhen. In addition, blood samples were collected from five healthy individuals as controls.RT-qPCR analysis was performed using these samples. The Longgang Central Hospital of Shenzhen Ethics Committee gave ethical approval for this research. Consent was given by all participating patients. RT-qPCR was employed to verify the expression levels of the biomarkers. Extraction of total RNA was carried out from 10 samples using TRIzol (Ambion, Austin, USA). The total RNA was converted into cDNA utilizing the SureScript First-strand cDNA Synthesis Kit (Servicebio, Wuhan, China), and RT-qPCR was carried out with the 2x Universal Blue SYBR Green qPCR Master Mix (Servicebio, Wuhan, China). Supplementary Table S2 contains the PCR primer sequences. GAPDH served as the internal control, with biomarker expression levels calculated using the 2^−ΔΔCt^ method (Livak and Schmittgen, 2001). Finally, the PCR results were imported into Graphpad for statistics and plotting. This study was approved by the Longgang Central Hospital of Shenzhen Ethics Committee. All participants provided written informed consent before sample collection, and all procedures adhered to the principles of the Declaration of Helsinki.

### 2.10 Statistical analysis

Statistical computations were executed in R (v 4.3.1). Group differences were evaluated using the Wilcoxon test. For RT-qPCR comparisons, the t-test was used, and statistical significance was considered to be less than 0.05.

### 2.11 Ethics approval and consent to participate

I certify that the research study titled [Identification and experimental validation of demethylation-related genes in diabetic nephropathy] has been approved by the relevant ethics committee or institutional review board (IRB).The approval number and date of approval are as follow: [2024ECYJ122]and [13 November 2024].

## 3 Results

This section systematically presented research findings from candidate gene screening to biomarker validation. First, based on differential expression analysis, we identified 161 candidate genes potentially associated with the pathogenesis of diabetic kidney disease (DKD). These genes were predominantly enriched in key biological processes such as inflammatory immune responses and tissue remodelling. Subsequently, we employed multiple machine learning methods to screen and select five key genes from this cohort; further expression validation and ROC curve analysis ultimately confirmed CXCL2 and MLF1 as core biomarkers with diagnostic potential. We then conducted in-depth investigations into the functional characteristics of these two biomarkers, their involvement in immune regulatory networks, and their potential therapeutic targets. Finally, all research findings were experimentally validated, confirming the expression levels of the biomarkers in independent samples.

### 3.1 Screening and analysis of enrichment patterns in candidate genes

**FIGURE 1:**
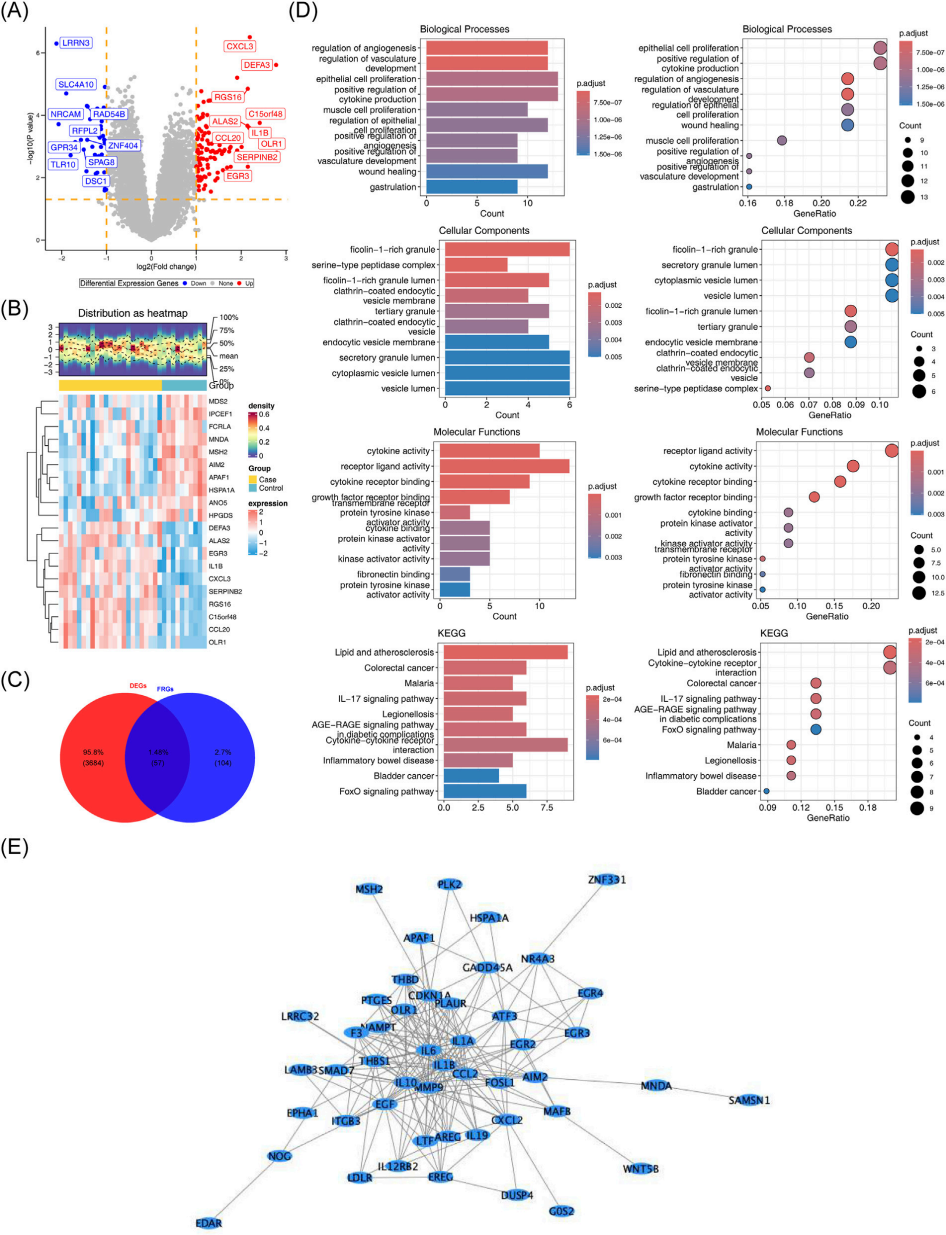
Screening and analysis of enrichment patterns in candidate genes. **(A)** Volcano plot of differentially expressed genes (DEGs) in diabetic nephropathy (DN). The top 10 up- and downregulated genes are shown. **(B)** Heatmap of DEGs between sample groups. Red: Case group Blue: control group. **(C)** Venn diagram showing the intersection of DEGs and demethylation-related genes (D-RGs). 57 candidate genes were detected. **(D)** Functional enrichment analysis of candidate genes. The depth of color reflects the adjusted P-value, with darker colors indicating smaller P-values. **(E)** Protein-protein interaction (PPI) network of candidate genes. The nodes in the graph represent specific proteins, while the edges indicate their interactions with each other.

GSE142153 identified 161 DEGs, of which 106 were upregulated genes and 55 were downregulated genes (Figures 1A,B). The DEGs and D-RGs were selected to be intersected, and 57 candidate genes were detected (Figure 1C). A total of 812 Gene Ontology (GO) biological functions were identified, including 758 biological processes (BP), 22 cellular components (CC), and 32 molecular functions (MF) (Figure 1D). In BP, these candidate genes were primarily associated with regulation of angiogenesis, regulation of vasculature development and epithelial cell proliferation (Supplementary Table S3); In CC, these candidate genes were mainly involved in ficolin-1-rich granule, serine-type peptidase complex and ficolin-1-rich granule lumen (Supplementary Table S4); In MF, these candidate genes were mainly involved in cytokine activity, receptor ligand activity and cytokine receptor binding (Supplementary Table S5). KEGG identified a total of 65 functional pathways that were enriched, specifically: lipid and atherosclerosis, colorectal cancer, and malaria. The PPI network constructed based on candidate genes contained 207 interactions of 48 proteins; there were nine proteins that did not interact with other proteins. Notably, the IL6, IL1B, and MMP9 protein interacts with most of the proteins in the network (Figure 1E) (Supplementary Table S6).

### 3.2 *CXCL2* and *MLF1* as the biomarkers

**FIGURE 2:**
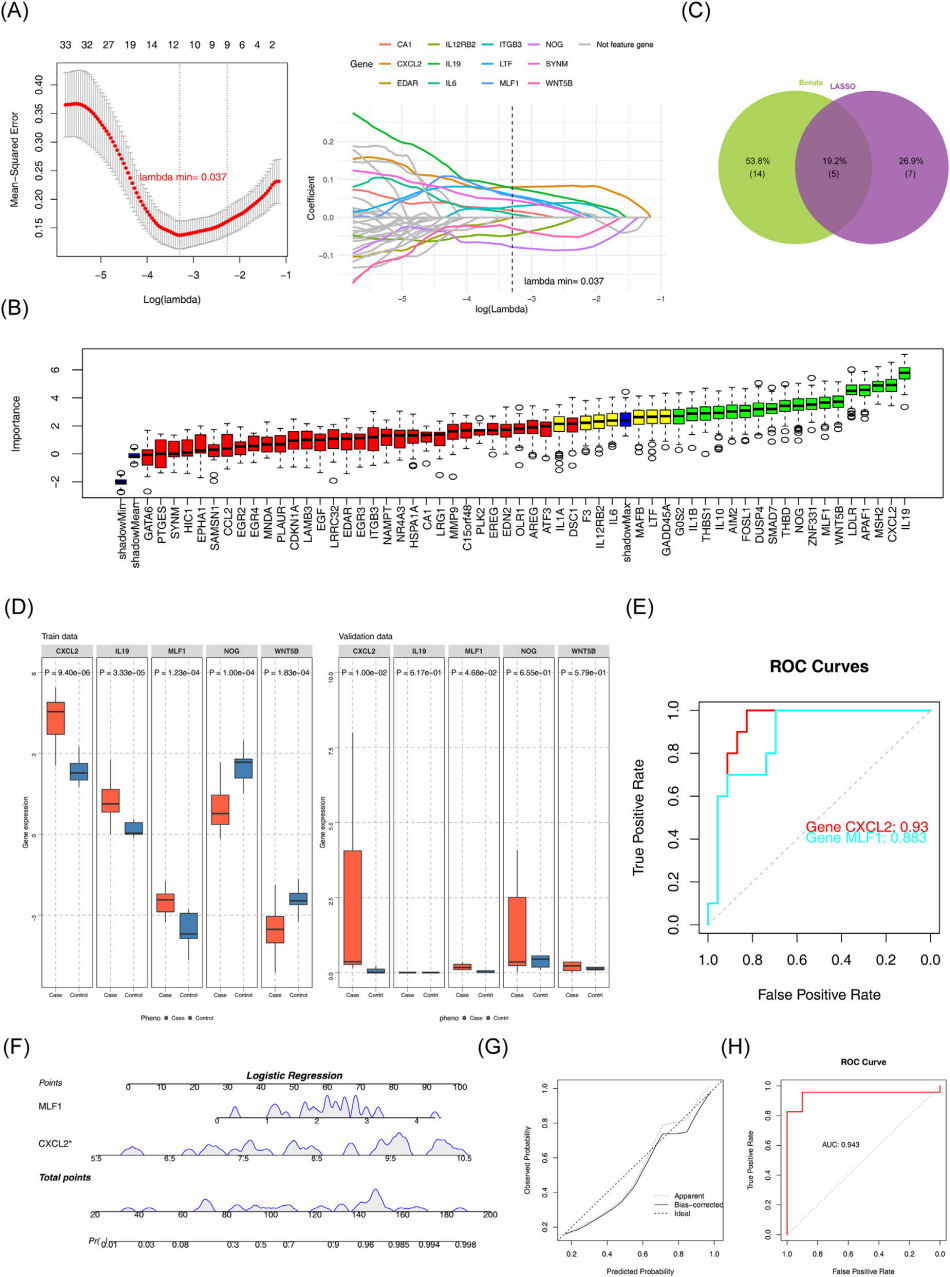
Identification of biomarkers **(A)** LASSO regression model and cross-validation results. **(B)** Importance plot for the LASSO regression model. **(C)** Feature selection results from Boruta and LASSO. **(D)** Gene expression comparison between case and control groups. **(E)** Receiver Operating Characteristic (ROC) curves for gene performance. **(F)** Logistic regression analysis for candidate gene prediction. **(G)** Calibration plot for the logistic regression model. **(H)** Receiver Operating Characteristic (ROC) curve for logistic regression model.

The LASSO algorithm selected optimal candidate genes based on minimum error criteria (lambda.min = 0.037), leading to a total of 12 candidate characterization genes 1 (Figure 2A). Furthermore, the Boruta algorithm identified 18 candidate characterization genes 2 (Figure 1B). The candidate characterization genes one and candidate characterization genes two were selected to be intersected, then five key genes were acquired: *CXCL2*, *IL19*, *MLF1*, *NOG*, and *WNT5B* (Figure 2C). The key genes expression was examined in DN patients, as well as in normal samples in GSE142153 (p < 0.05) and validated in the GSE154881 (p < 0.05). As shown in Figure (Figure 2D), the results indicated that *CXCL2* and *MLF1* exhibited significantly high expression in DN samples and the same trends in the two datasets. Therefore, we chose *CXCL2* and *MLF1* as biomarkers for this study. The ROC curves were analyzed for the two biomarkers. Analysis revealed that the AUC of the *CXCL2* and *MLF1* were greater than 0.7 in the GSE142153 (Figure 2E). Based on the two biomarkers identified as previously described, a nomogram was developed using the GSE142153, the higher total scores in the nomogram indicated a greater risk of DN development (Figure 2F), The calibration curve’s close alignment with the ideal curve suggested that the nomogram was more accurate (Figure 2G), and the results in ROC curves showed the nomogram had good predictive effects in GSE142153 (Figure 2H), with the model’s AUC values was 0.943.

### 3.3 GSEA results for biomarkers

**FIGURE 3:**
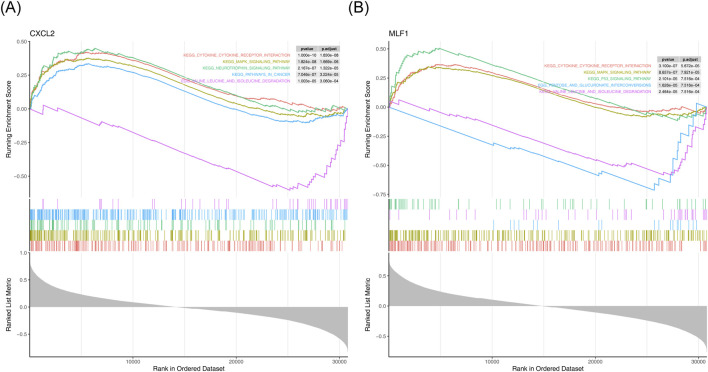
GSEA results for biomarkers **(A)** Enrichment analysis of *CXCL2* in KEGG pathways. **(B)** Enrichment analysis of *MLF1* in KEGG pathways.

GSEA results showed that *CXCL2* enriched 50 functional pathways (Figure 3A) (Supplementary Table S7). MLF1 was enriched for a total of 30 functional pathways (Figure 3B) (Supplementary Table S8). The most significantly enriched pathways for both *CXCL2* and *MLF1* were kegg cytokine cytokine receptor interaction (*CXCL2*: NES = 2.057, Adjust P < 0.05; *MLF1*: NES = 1.840, Adjust P < 0.05) and kegg mapk signaling pathway (C*XCL2*: NES = 1.858, Adjust P < 0.05; *MLF1*: NES = 1.757, Adjust P < 0.05).

### 3.4 A total of five differential immune cells

**FIGURE 4:**
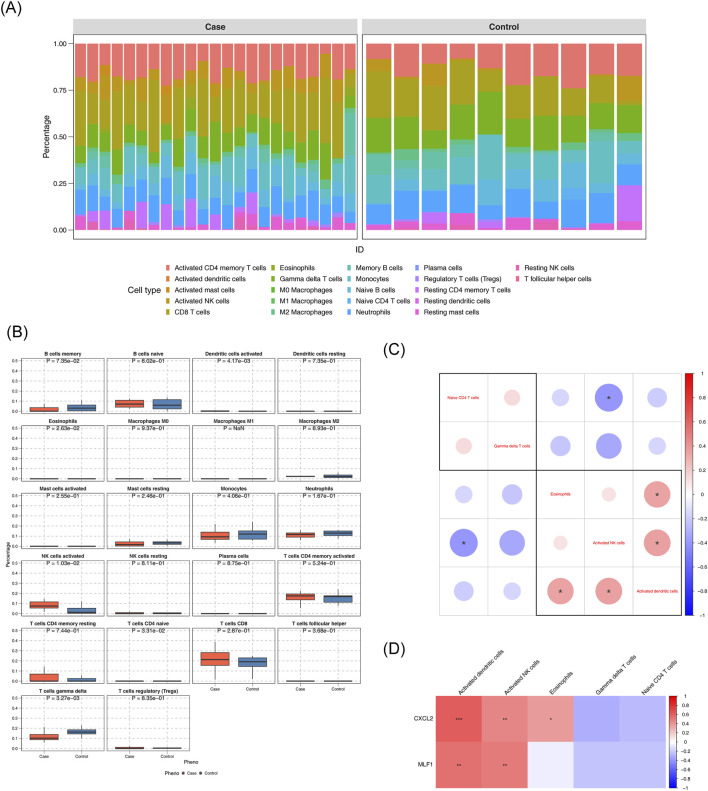
Analysis of the immune infiltration **(A)** Immune cell composition in diabetic nephropathy (DN) and control groups. **(B)** Comparison of immune cell percentages between case and control groups. **(C)** Differential expression of immune cell markers in DN. **(D)** Correlation of immune cell markers with gene expression.

Stacked plots illustrated the comparative percentages of 22 immune cells (Figure 4A). Comparative analysis of immune cell infiltration revealed statistically significant differences in activated dendritic cells, eosinophils, activated NK cells, naive CD4^+^ T cells, and gamma delta T cells between DN and control groups. (Figure 4B). The correlation analysis between various differential immune cells was conducted, revealing a remarkable positive association between NK cells activated and dendritic cells activated (cor = 0.365, p < 0.05). The correlation analysis between various differential immune cells was conducted, unveiling a remarkable negative association between naive CD4^+^ T cells and NK cells activated (cor = - 0.4, p < 0.05) (Figure 4C) (Supplementary Table S9). Differential immune cell and biomarker correlation were a remarkable positive association *CXCL2* and NK cells activated (cor = 0.476,p value <0.05), However, the most significant negative correlation was *CXCL2* and gamma delta T cells (cor = - 0.296, p < 0.05); The strongest positive association was *MLF1* and NK cells activated (cor = 0.502, p < 0.05). However, the most significant negative correlation was *MLF1* and naive CD4^+^ T cells (cor = − 0.219, p < 0.05) (Figure 4D) (Supplementary Table S10).

### 3.5 Subcellular localization, chromosome localization, renal function correlation, and disease relevance of biomarkers

**FIGURE 5:**
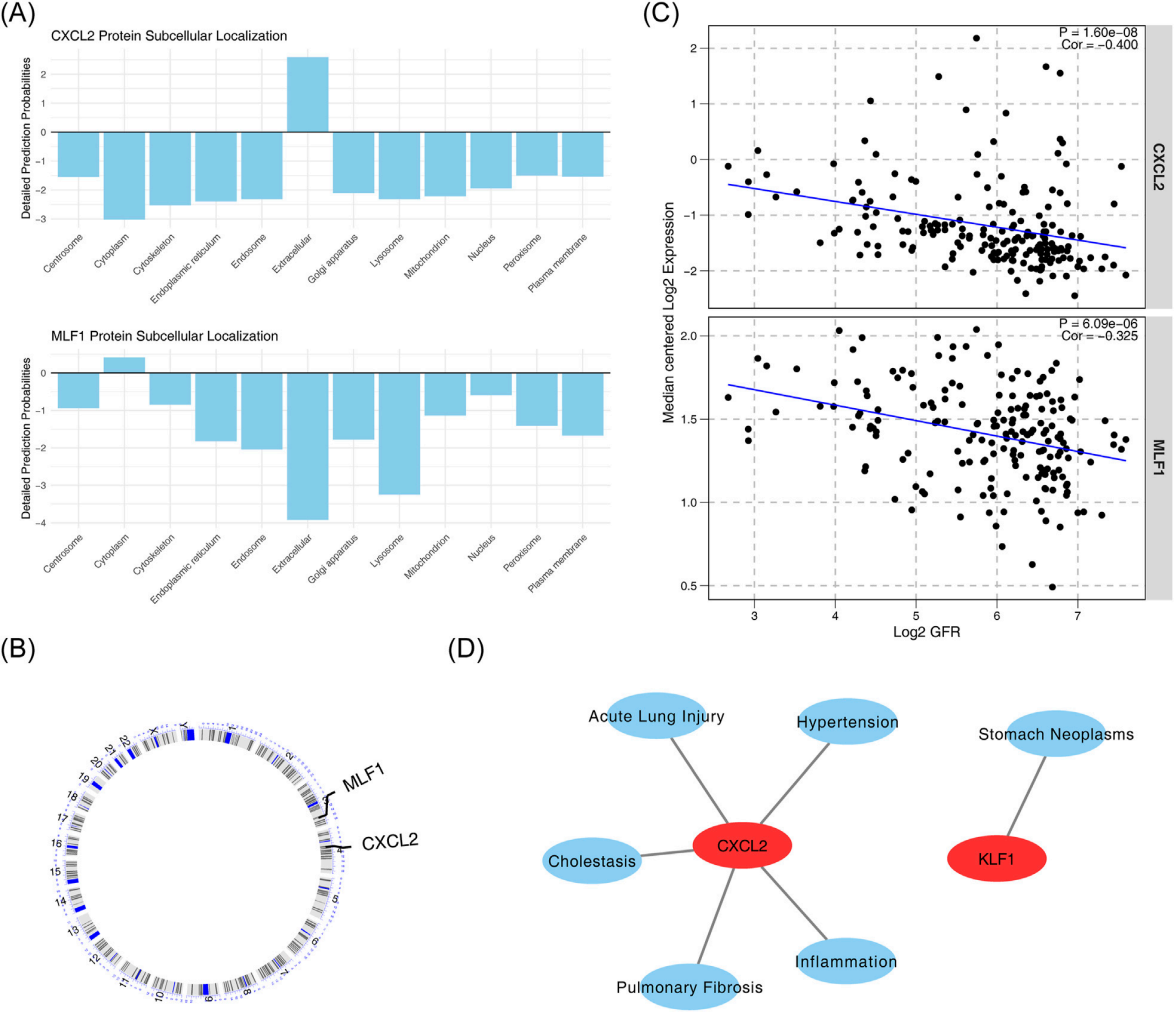
Subcellular localization, chromosome localization, renal function correlation, and disease relevance of biomarkers. **(A)** Subcellular localization of *CXCL2* and *MLF1* proteins. **(B)** Correlation between *CXCL2* and *MLF1* protein expression. **(C)** Correlation between *CXCL2* and *MLF1* expression with Log2 GFR. **(D)** Association of *CXCL2* with various diseases and conditions.

Subcellular localization analysis of biomarkers showed that *CXCL2* was more expressed in the extracellular space, while *MLF1* was more expressed in the cytoplasm (Figure 5A). Chromosome localization analysis of biomarkers showed that *CXCL2* was localized to chromosome 4, and *MLF1* was localized to chromosome 3 (Figure 5B). The correlation analysis between biomarkers and kidney function yielded statistically significant negative results between *CXCL2* expression levels and GFR values (cor = −0.400, p < 0.05), and statistically significant negative association between *MLF1* expression levels and GFR values (cor = −0.325, p < 0.05) (Figure 5C). Diseases that were significantly associated with *CXCL2* include cholestasis, inflammation, acute lung injury, pulmonary fibrosis and hypertension, and those that were significantly associated with MLF1 was stomach neoplasms (Figure 5D).

### 3.6 Regulatory networks for biomarkers

**FIGURE 6:**
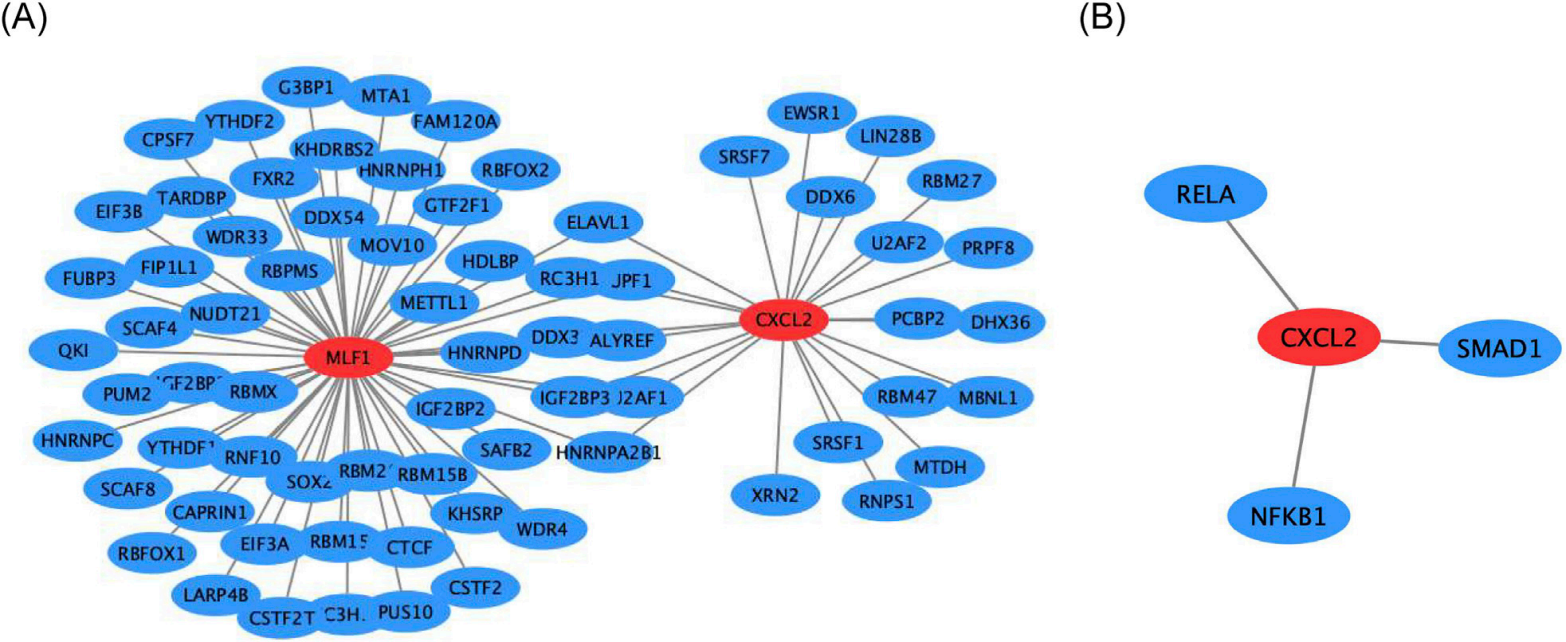
Regulatory networks for biomarkers **(A)** Gene interaction network of *CXCL2* and *MLF1*. **(B)** Gene interaction network of *CXCL2* with *RELA, SMAD1*, and *NFKB1*.

Firstly, the mRNA-RBP, the study results indicated that five RNA binding proteins were significantly enriched in both biomarkers, including *DDX3X*, *ELAVL1*, *HNRNPA2B1*, *IGF2BP3*, *RC3H1*, *U2AF1*, and *UPF1* (Figure 6A). In addition, the mRNA-TF network was constructed *CXCL2* obtained 3 TFs acting on biomarkers, including *RELA*, *NFKB1*, and *SMAD1* (Figure 6B).

### 3.7 A total of 172 target drugs

**FIGURE 7:**
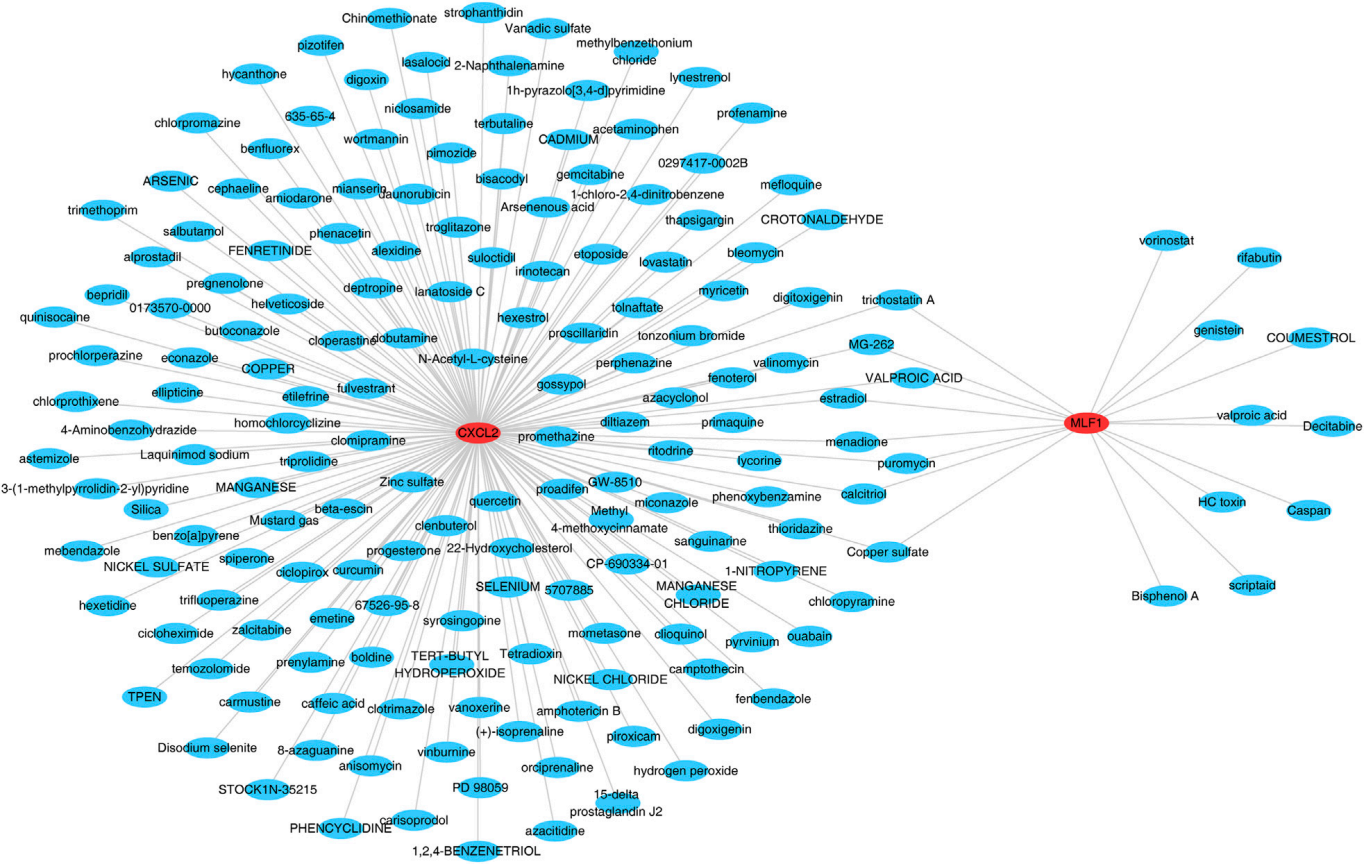
Gene interaction network of *CXCL2* and *MLF1*.

The database was searched for the 172 target drugs of the two biomarkers, and both biomarkers predicted nine drugs, including valproic acid, trichostatin A, puromycin, *MG-262*, menadione, estradiol, copper sulfate, and calcitriol (Figure 7) (Supplementary Table S11).

### 3.8 Confirmation of biomarker expression

**FIGURE 8:**
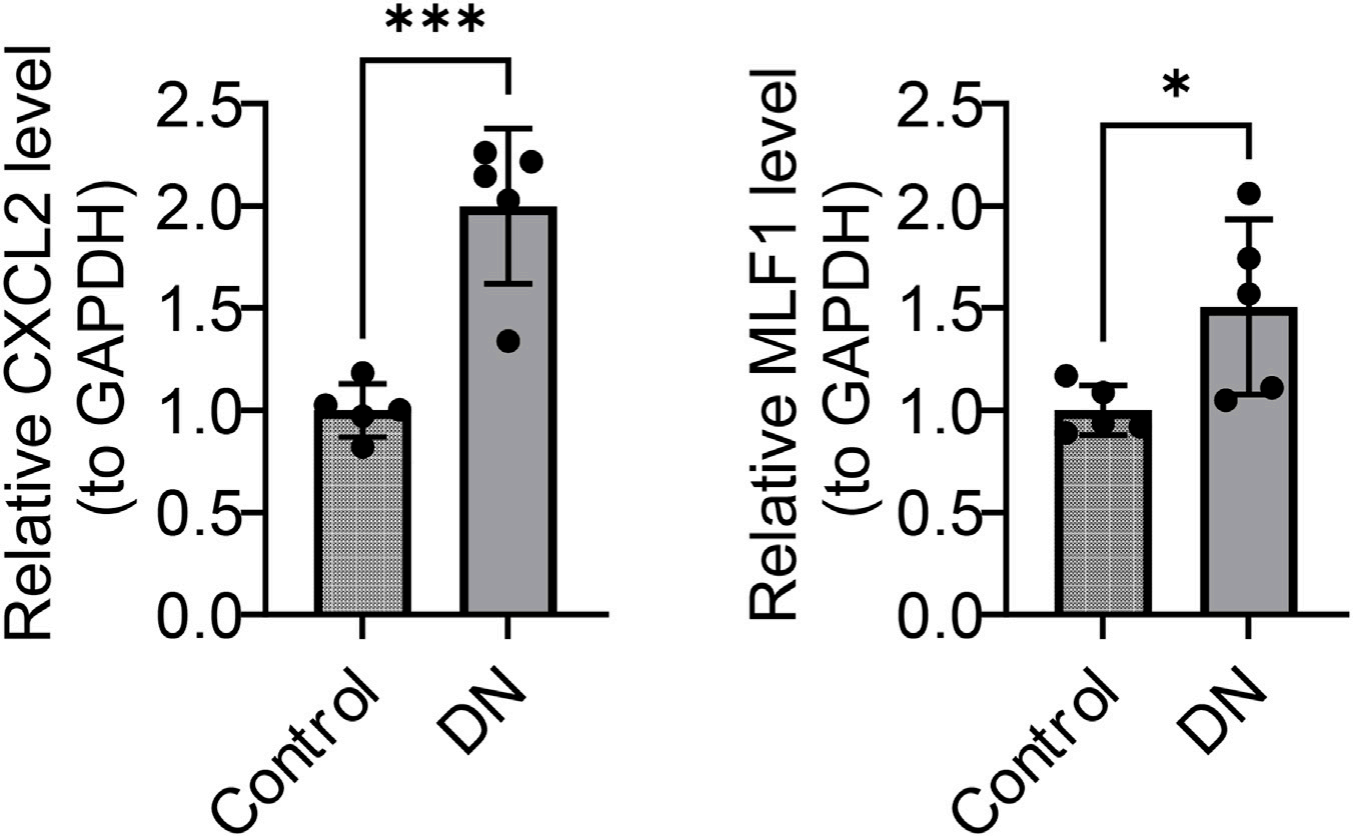
Expression levels of *CXCL2* and *MLF1* in control and DN samples (Mean ± SD).

RT-qPCR was employed to quantify and compare the expression levels of *CXCL2* and *MLF1* between the DN and normal groups (Figure 8; Table 1). The DN group exhibited significantly elevated levels of both *CXCL2* and *MLF1* compared to the normal groups (p < 0.05).

**TABLE 1:** Detection of CXCL2 and MLF1 expression levels in control and DN samples by RT-PCR (Mean ± SD).

Gene	Control	DN	P-value
CXCL2	1 ± 0.1292	1.998 ± 0.3798	0.0005
MLF1	1 ± 0.1214	1.5061 ± 0.4286	0.0347

## 4 Discussion

DN, one of the most severe microvascular complications of diabetes, has emerged as a primary cause of end-stage renal disease worldwide, with its pathogenesis involving complex interactions among glucose metabolism disorders, oxidative stress, inflammatory responses, and epigenetic dysregulation (Jin et al., 2023; Thipsawat, 2021; Samsu, 2021). Through integrated multi-omics analysis and machine learning approaches, this study has successfully identified *CXCL2* and *MLF1* as novel diagnostic biomarkers for DN, laying a crucial foundation for developing innovative diagnostic and therapeutic strategies.*CXCL2* (C-X-C Motif Chemokine Ligand 2) is a small protein that is secreted by cells such as active monocytes, neutrophils, and macrophages. It belongs to the CXC chemokine family and is also known as *MIP-2*, *GRO-Beta*, or *GR O -2* (Guo et al., 2020). In terms of amino acid sequence, it has 90% similarity to the related chemokine *CXCL1* (Lin et al., 2021). It binds to the *CXCR2* receptor and is located on human chromosome 4 (51). *CXCL2* exhibits chemotactic activity toward neutrophils, polymorphonuclear leukocytes, and hematopoietic stem cells, facilitating their recruitment to sites of inflammation or injury (Guo et al., 2020; Cai et al., 2023). In terms of immune regulation, *CXCL2* is involved in a variety of immune responses, including wound healing, cancer metastasis, and angiogenesis. As a protein encoded by an oncogene, *CXCL2* promotes angiogenesis and plays a critical role in the initiation, progression, and metastasis of tumors (Cai et al., 2023). *CXCL2* induces a dose-dependent increase in the migration of colorectal tumor cells *in vitro*. Silencing *CXCL1* and *CXCL2* downregulates multiple metastasis-promoting genes and inhibits the metastatic potential of breast cancer cells (Cai et al., 2023). Regarding blood regulation, *CXCL2* inhibits the proliferation of hematopoietic progenitor cells while activating ERK to enhance the proliferation of bone marrow-derived macrophage precursor cells (BMMS). The knockout of *CXCL2* suppresses both proliferation and ERK activation in BMMS. In models of platelet-activating factor-induced shock and intestinal injury, lipopolysaccharide (LPS) induces *CXCL2* expression, thereby promoting neutrophil migration (Guo et al., 2020). In acute lung injury, CXCR2 ligands (including *CXCL1/2/3*) exhibit chemotactic activity toward polymorphonuclear leukocytes (Zhang et al., 2023). CXCL2 represents a novel therapeutic target for inflammatory bone destruction diseases. Recent research employs *CXCL2* chemokine as an intermediary in sepsis treatment (Zhang et al., 2023). The expression and significance of *CXCL2* in tumors have garnered significant attention, potentially serving as a diagnostic marker for various tumors, a detection factor for tumor recurrence, and a promising therapeutic target. Studies indicate that during tolerance induction, lymphocyte circulation can modulate new therapies such as autoimmunity management, transplantation acceptance, and tumor suppression. In our study, *CXCL2* levels were high in DN patients. This fits with earlier work showing that *CXCL2* can worsen kidney scarring and stress (He et al., 2022). We also saw that *CXCL2* is involved in the *MAPK* pathway. This pathway is known to cause kidney cell death and inflammation when blood sugar is high (Ambujakshan and Sahu, 2025). Moreover, higher *CXCL2* levels are linked with lower kidney function (GFR), which supports its use as a marker for kidney decline (Nguyen et al., 2022).

*MLF1* (Myeloid leukemia factor 1) is a protein that works in the cell nucleus to control gene activity. It has a signal that sends it to the nucleus, and it can bind to DNA. *MLF1* is found in many cells, including blood, nerve, and cancer cells. *MLF1* functions as either a transcriptional repressor or activator, thereby participating in the regulation of various gene transcriptions. It modulates gene expression levels through interactions with DNA or other transcription factors (Li Z. et al., 2023). MLF1 exhibits DNA-binding capability and can recognize and bind to specific DNA sequences, directly impacting the transcriptional activity of target genes. Furthermore, *MLF1* interacts with other transcription factors, cofactors, or chromatin-associated proteins to form a complex regulatory network (Lv et al., 2025; Vinopalová et al., 2024). These interactions may influence *MLF1*’s functionality by altering its DNA-binding affinity, transcriptional activity, or subcellular localization. *MLF1* plays a pivotal role in cell proliferation and differentiation by influencing cell cycle progression and promoting or inhibiting cell proliferation. Additionally, *MLF1* contributes to determining the differentiation direction of cells, thereby shaping their functional and phenotypic characteristics (Tang et al., 2023). Studies have shown that *MLF1* plays a critical role in tumor initiation and progression across various cancer types. In certain types of tumors, alterations in *MLF1* expression levels have been observed, which may correlate with tumor malignancy, prognosis, and therapeutic outcomes (Shimosato et al., 2025). Modulating the expression or function of *MLF1* could potentially provide novel insights and strategies for tumor treatment (Huang et al., 2020). Other work has shown that *MLF1* plays a role in aging and cell regulation (Li Z. et al., 2023). Our study shows that *MLF1* is high in DN. It is linked to the enhanced function of natural killer (NK) cells and dendritic cells. This suggests that MLF1 may connect changes in gene methylation to problems in immune control (Lv et al., 2025). We also found that both *MLF1* and *CXCL2* are active in the *MAPK* pathway, which means they may work together to worsen DN (53). As a promising target for tumor therapy, *MLF1* has garnered significant attention. Ongoing research aims to elucidate the precise mechanisms underlying *MLF1*’s role in tumorigenesis and progression, as well as to develop targeted therapeutic approaches. Regulating the expression level or function of *MLF1* may offer new gene therapy strategies for certain genetic disorders or tumors, while also providing potential drug screening targets.

Our differential immune cell correlation analysis revealed significant positive correlations between activatedNK cells and activated dendritic cells (DCs), whereas naive CD4^+^ T cells showed a pronounced negative correlation with activated NK cells. In biomarker-immune cell correlation assessments: *CXCL2* exhibited strong positive correlations with activated NK cells but negative correlations with γδ T cells; *MLF1* demonstrated the strongest positive correlation with activated NK cells and the most pronounced negative correlation with naive CD4^+^ T cells. Mo Li et al. reported that miR-218 ameliorates DN by targeting IKK-β and suppressing NF-κB-mediated inflammation, evidenced by reduced levels of TNF-α, IL-6, IL-1β, and MCP-1 upon miR-218 overexpression (Li et al., 2020). Concurrently, Qianqian Han et al. identified elevated CD4^+^ T cell infiltration density as a predictor of severe nephropathic lesions and renal function decline in DN patients (Han et al., 2024). These findings delineate a “dual track” immunopathogenic model for diabetic nephropathy (DN): On one hand, chemokines such as *CXCL2* drive the activation of innate immune components including NK cells; on the other hand, they induce functional dysregulation of CD4^+^ T cells. These dual immunologic perturbations converge through NF-κB signaling pathways, culminating in chronic renal inflammation and fibrosis. This comprehensive understanding not only deepens mechanistic insights into DN pathogenesis but also informs the development of precision immunomodulatory strategies. Future therapeutic exploration may prioritize combined interventions targeting NK cell activation thresholds and T cell functional recalibration to achieve synergistic efficacy.

Our study used several methods to find DN markers. We compared gene expression between DN patients and healthy controls and then combined this with demethylation data. This allowed us to screen out key candidate genes. We then applied LASSO and Boruta algorithms to pick the best markers. Our work shows that *CXCL2* and *MLF1* link gene methylation changes with inflammation. We built a prediction model (nomogram) that had a high AUC of 0.943. This method supports recent ideas that use both gene and immune data to study kidney disease (Jin et al., 2023; Bhayana et al., 2025; Shapiro et al., 2023).

We also found 172 drugs that might target *CXCL2* and *MLF1*. Some drugs, such as valproic acid and trichostatin A, have been shown to reduce kidney damage. For example, trichostatin A can lower kidney scarring and inflammation in animal models (Gupta et al., 2023). The link between *CXCL2/MLF1* levels and immune cell changes suggests that targeting these markers may alter the immune environment in DN, which could lead to new treatments (Chen Y. et al., 2024).

Our study has some limitations. We used blood samples to study gene expression. This may not capture all the changes that occur in the kidney. This is a common issue in DN marker studies (Nguyen et al., 2022). We confirmed the high levels of *CXCL2* and *MLF1* by RT-qPCR, but more experiments are needed to prove that these genes cause DN. Future work should use animal models or CRISPR-based methods to study these genes further. Other studies, such as those on TET1 in diabetic eye disease, show that such methods can be useful (Tan et al., 2021). Additionally, the datasets used in this study suffers from class imbalance, which may adversely affect the stability of machine learning models. Although we employed multiple strategies such as cross-validation and independent dataset validation during the research, future studies are still required to further verify our findings in larger and more balanced datasets.

In short, our study shows that *CXCL2* and *MLF1* are promising markers for diagnosing DN. They link gene methylation changes with immune and inflammatory pathways. Our use of machine learning and various data methods provides a strong way to find markers and treatment targets. Future research should confirm these results in larger groups and study the detailed roles of these genes in DN.

## Data Availability

The datasets analyzed for this study can be found in the Gene Expression Omnibus (GEO) database at https://www.ncbi.nlm.nih.gov/geo/. Specifically, the accession numbers are GSE142153 and GSE154881.

## Supplementary material

Supplementary Table 1 3741 demethylation-related genes.

Supplementary Table 2 Summary Table of PCR primer sequences.

Supplementary Table 3 Summary Table of GO analysis.

Supplementary Table 4 Summary Table of GO analysisi cellular components.

Supplementary Table 5 Summary Table of GO analysisi molecular functions.

Supplementary Table 6 Summary Table of KEGG analysis.

Supplementary Table 7 Summary Table of f GSEA Results for CXCL2.

Supplementary Table 8 Summary Table of GSEA Results for MLF1.

Supplementary Table 9 Summary Table of correlation analysis between various differential immune cells.

Supplementary Table 10 Summary Table of Differential immune cell and biomarker correlation.

Supplementary Table 11 Summary Table of target drugs.

Supplementary Image 1 Mechanism diagram of diabetic nephropathy.

## Abbreviations

DN, Diabetic Nephropathy; YSXZF, Yi-Shen-Xie-Zhuo Formula; TCM, Traditional Chinese Medicine.
