# Supplementary figures and images for: Identification and experimental validation of demethylation-related genes in diabetic nephropathy
Source: Front Genet. 2025 Nov 26;16:1675592. doi: 10.3389/fgene.2025.1675592 (PMC12688277; doi:10.3389/fgene.2025.1675592)

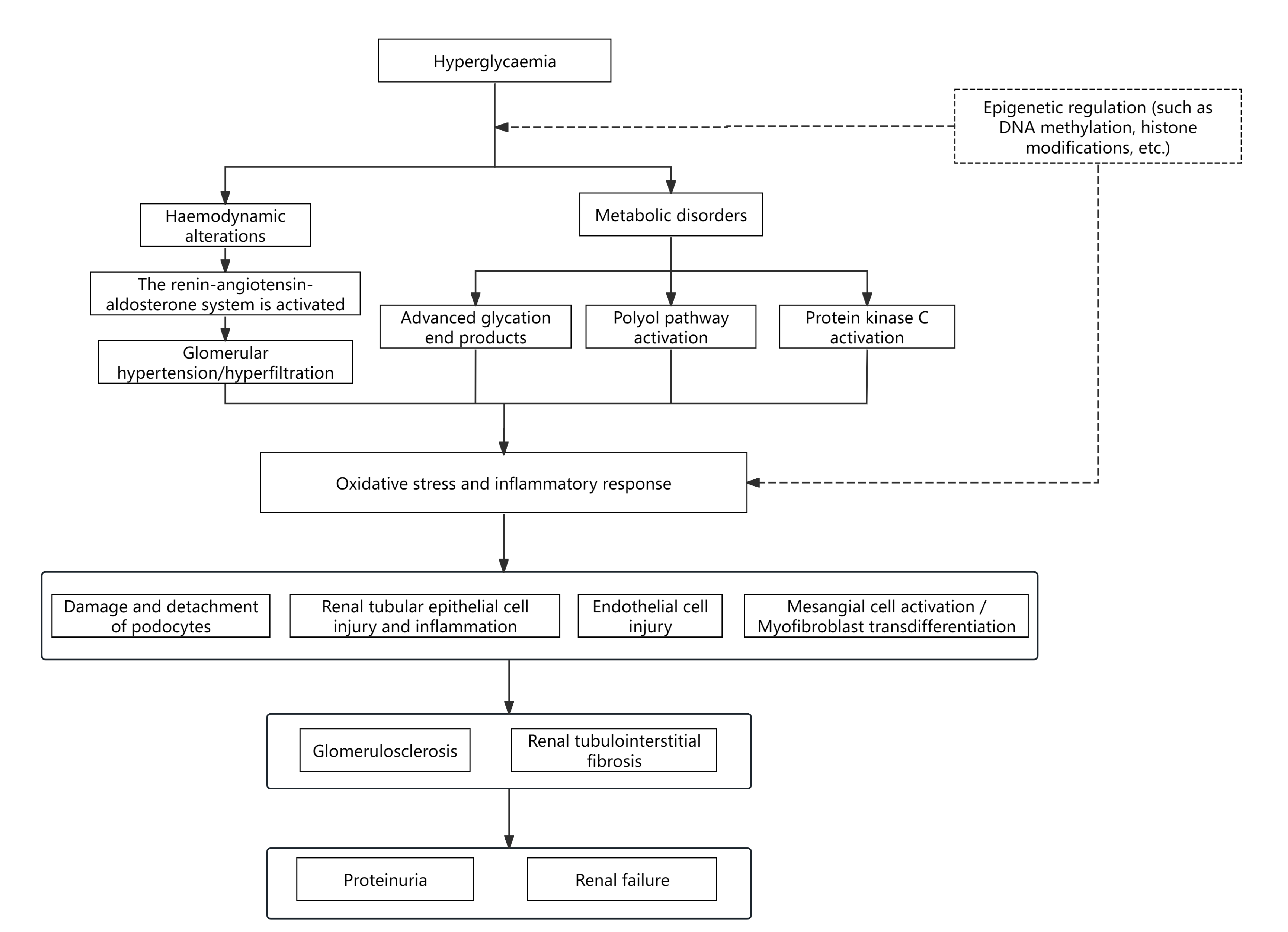

Supplement: Supplementary file 3 [file Image1.tif]
